# CRISPR‐LbCas12a‐mediated modification of citrus

**DOI:** 10.1111/pbi.13109

**Published:** 2019-04-10

**Authors:** Hongge Jia, Vladimir Orbović, Nian Wang

**Affiliations:** ^1^ Department of Microbiology and Cell Science Citrus Research and Education Center Institute of Food and Agricultural Sciences (IFAS) University of Florida Lake Alfred FL USA; ^2^ Citrus Research and Education Center IFAS University of Florida Lake Alfred FL USA; ^3^ China‐USA Citrus Huanglongbing Joint Laboratory (A joint laboratory of The University of Florida’s Institute of Food and Agricultural Sciences and Gannan Normal University) National Navel Orange Engineering Research Center Gannan Normal University Ganzhou Jiangxi China

**Keywords:** Cas12a, citrus, Cpf1, CRISPR, genome editing

## Abstract

Recently, CRISPR‐Cas12a (Cpf1) from *Prevotella* and *Francisella* was engineered to modify plant genomes. In this report, we employed CRISPR‐LbCas12a (LbCpf1), which is derived from *Lachnospiraceae bacterium ND2006*, to edit a citrus genome for the first time. First, LbCas12a was used to modify the *CsPDS* gene successfully in Duncan grapefruit via Xcc‐facilitated agroinfiltration. Next, LbCas12a driven by either the 35S or Yao promoter was used to edit the PthA4 effector binding elements in the promoter (EBE_P__thA4_‐CsLOBP) of *CsLOB1*. A single crRNA was selected to target a conserved region of both Type I and Type II CsLOBPs, since the protospacer adjacent motif of LbCas12a (TTTV) allows crRNA to act on the conserved region of these two types of CsLOBP. *CsLOB1* is the canker susceptibility gene, and it is induced by the corresponding pathogenicity factor PthA4 in *Xanthomonas citri* by binding to EBE_P__thA4_‐CsLOBP. A total of seven 35S‐LbCas12a‐transformed Duncan plants were generated, and they were designated as #D_35_s1 to #D_35_s7, and ten Yao‐LbCas12a‐transformed Duncan plants were created and designated as #D_yao_1 to #D_yao_10. LbCas12a‐directed EBE_P__thA4_‐CsLOBP modifications were observed in three 35S‐LbCas12a‐transformed Duncan plants (#D_35_s1, #D_35_s4 and #D_35_s7). However, no LbCas12a‐mediated indels were observed in the Yao‐LbCas12a‐transformed plants. Notably, transgenic line #D_35_s4, which contains the highest mutation rate, alleviates XccΔpthA4:dCsLOB1.4 infection. Finally, no potential off‐targets were observed. Therefore, CRISPR‐LbCas12a can readily be used as a powerful tool for citrus genome editing.

## Introduction

Genome editing is a powerful tool for increasing plant yields, improving food quality, enhancing crop disease resistance and developing new cultivars to meet market needs (Begemann *et al*., 2017). At present, several strategies are being exploited to edit plant genomes, including CRISPR‐Cas, meganucleases, TALENs and zinc finger nucleases (Martín‐Pizarro and Posé, 2018). Among them, clustered regularly interspaced short palindromic repeat (CRISPR)‐mediated genome editing is the most attractive, owing to its comparatively easier and less expensive implementation (Islam, 2018). To date, CRISPR‐SpCas9, which is derived from *Streptococcus pyogenes*, has been widely used to modify the genomes of a variety of organisms. However, one major drawback associated with the CRISPR‐SpCas9 system is its off‐target effects (Fu *et al*., 2013), which has raised concerns and limited its adoption. Recently, CRISPR‐Cas12a from *Prevotella* and *Francisella*, a class II/type V CRISPR nuclease, has been employed as an alternative system for genome editing, and notably, it is reported to have fewer off‐targets in comparison with Cas9 (Kim *et al*., 2016; Kleinstiver *et al*., 2016).

CRISPR‐Cas12a has several unique features distinct from those of CRISP‐SpCas9 (Zetsche *et al*., 2015, 2017), as follows. (i) The canonical protospacer adjacent motif (PAM) of CRISPR‐Cas12a is TTTV (V=A, C and G), which is located at the 5′ end of the target site, whereas the CRISPR‐SpCas9 PAM is NGG, which is located at the 3′ end of the target site. (ii) CRISPR‐Cas12a requires a 43 nt crRNA, and CRISPR‐SpCas9 requires ~100 nt gRNA. (iii) CRISPR‐Cas12a generates 5′ staggered ends distal from the PAM, while CRISPR‐SpCas9 generates blunt ends 3 bp upstream of the PAM. (iv) Cas12a has both DNase activity and RNase activity, which is useful for multiplexed genome editing (Zetsche *et al*., 2017). These complementary properties make the CRISPR‐Cas12a an indispensable genome‐editing tool. CRISPR‐Cas12a was first successfully employed to edit the mammalian genome (Zetsche *et al*., 2015). Since then, CRISPR‐Cas12a has been successfully used to modify other organisms, such as plants, *Drosophila* and zebrafish (Endo *et al*., 2016; Moreno‐Mateos *et al*., 2017; Port and Bullock, 2016). To date, *Acidaminococcus* sp. BV3L6 Cas12a (AsCas12a), *Francisella novicida* Cas12a (FnCas12a) and *Lachnospiraceae bacterium ND2006* Cas12a (LbCas12a) have been used to edit the genomes of crop and model plants, including green alga, rice, soybeans and tobacco (Begemann *et al*., 2017; Endo *et al*., 2016; Ferenczi *et al*., 2017; Hu *et al*., 2017; Kim *et al*., 2017; Tang *et al*., 2017; Wang *et al*., 2017a,b,c; Xu *et al*., 2016; Yin *et al*., 2017), but not citrus. In addition, the performances of the three Cas12a homologs are different. LbCas12a reportedly performs better than AsCas12a in rice (Tang *et al*., 2017).

Citrus is an important fruit crop around the world, and it faces many abiotic and biotic stresses such as citrus canker and Huanglongbing disease (Ference *et al*., 2018; Wang and Trivedi, 2013; Wang *et al*., 2017a,b). To promote citrus breeding, CRISPR‐SpCas9 has been employed to edit the citrus genome in several reports (Jia and Wang, 2014a; Jia *et al*., 2016, 2017b; Peng *et al*., 2017; Zhang *et al*., 2017). *CsLOB1* is reportedly the canker susceptibility gene (Hu *et al*., 2014). To produce citrus varieties that are resistant to *Xanthomonas citri* subsp. citri (Xcc) infection, CRISPR‐SpCas9 was used in two studies to modify the PthA4 effector binding elements (EBEs) in the *CsLOB1* promoter (EBE_PthA4_‐CsLOBP; Jia *et al*., 2016; Peng *et al*., 2017). However, only the Type I EBE_PthA4_‐CsLOBP was modified in transgenic Duncan grapefruit, because no suitable sgRNAs can be designed to target both alleles of EBE_PthA4_‐CsLOBP owing to the single nucleotide polymorphisms in this region (Jia *et al*., 2016). Duncan grapefruit is a hybrid between the pummelo (*Citrus maxima*) and the sweet orange (*Citrus sinensis*; Velasco and Licciardello, 2014). Type I CsLOBP originates from sweet orange (Xu *et al*., 2013), and Type II CsLOBP comes from the pummelo (Wu *et al*., 2014). Therefore, one of the challenges of SpCas9‐mediated EBE_PthA4_‐CsLOBP modification is the fact that two types of CsLOBPs in Duncan grapefruits make a single sgRNA targeting infeasible for modifying two alleles. An alternative genome‐editing system that can be employed to edit two alleles of EBE_PthA4_‐CsLOBPs using a single sgRNA/crRNA would be helpful. CRISPR‐Cas12a recognizes a thymidine‐rich PAM site, TTTV, which commonly occurs in the promoter regions and the 5′ and 3′ UTRs (Moreno‐Mateos *et al*., 2017; Zetsche *et al*., 2015). Undoubtedly, it is worth testing whether CRISPR‐Cas12a can be employed to modify citrus to expand our biotechnology toolbox.

In this study, LbCas12a was employed to edit the Duncan grapefruit gene *CsPDS* via Xcc‐facilitated agroinfiltration. The results verified that LbCas12a could be harnessed to edit the citrus genome. Subsequently, using a single crRNA, EBE_PthA4_‐CsLOBP was successfully modified by LbCas12a in transgenic Duncan plants. Notably, one transgenic Duncan line could alleviate XccΔpthA4:dCsLOB1.4‐induced canker symptoms.

## Results

### Modification of *CsPDS* in Duncan grapefruit via the Xcc‐facilitated agroinfiltration of GFP‐p1380N‐35S‐LbCas12a‐crRNA‐cspds

**Figure 1:**
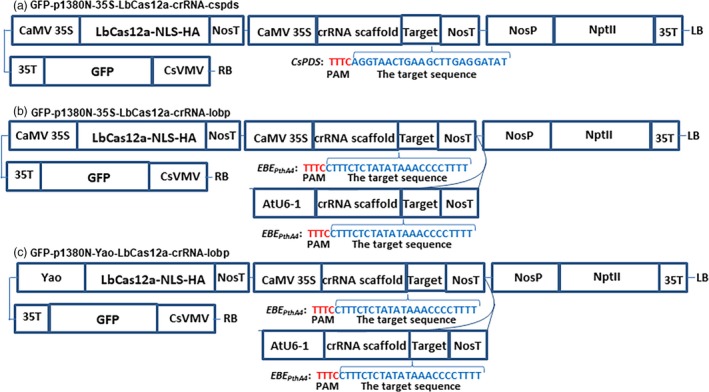
The Schematic diagram of binary vectors. (a) Schematic diagram of GFP‐p1380N‐35S‐LbCas12a‐crRNA‐cspds. A 23 bp crRNA was used to target the *CsPDS* coding region, which is located in the ninth extron. (b) Schematic diagram of GFP‐p1380N‐35S‐LbCas12a‐crRNA‐lobp. A 23 bp crRNA, driven by CaMV 35S and AtU6‐1, was employed to target EBE_P__thA4_‐CsLOBP. (c) A schematic diagram of GFP‐p1380N‐Yao‐LbCas12a‐crRNA‐lobp. A 23 bp sgRNA was designed to edit the EBE_P__thA4_‐CsLOBP. CsVMV, the cassava vein mosaic virus promoter; GFP, green fluorescent protein; CaMV 35S and 35T, the cauliflower mosaic virus 35S promoter and its terminator; AtU6‐1, *Arabidopsis* U6‐1 promoter; Yao, Yao promoter; LbCas12a‐NLS‐HA, the LbCas12a endonuclease containing nuclear location signal and HA tag at its C‐terminal; targets were highlighted in blue; PAM, protospacer adjacent motif, highlighted in red; crRNA scaffold, the CRISPR RNA scaffold; NosP and NosT, the nopaline synthase gene promoter and its terminator; NptII, neomycin phosphotransferase II; and LB and RB, the left and right borders of the T‐DNA region.

**Figure 2:**
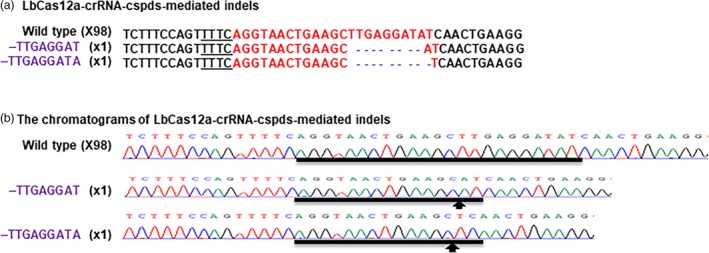
Targeted genome engineering in Duncan grapefruit using the CRISPR‐LbCas12a system. (a) Targeted mutations induced by GFP‐p1380N‐35S‐LbCas12a‐crRNA‐cspds in the *CsPDS* gene in Duncan grapefruit. The crRNA‐targeted *CsPDS* sequence is highlighted in red, and the indels are shown in purple. (b) CRISPR‐LbCas12a‐mediated indel chromatograms in the *CsPDS* gene. Mutations are indicated by arrows.

CaMV 35S‐SpCas9/CaMV 35S‐sgRNA and CaMV 35S‐SaCas9/CaMV 35S‐sgRNA were used to test the CRISPR‐Cas9 function through Xcc‐facilitated agroinfiltration (Jia and Wang, 2014a; Jia *et al*., 2017a). Therefore, CaMV 35S alone was used to drive both LbCas12a and crRNA in vector GFP‐p1380N‐35S‐LbCas12a‐crRNA‐cspds, which was harnessed for Xcc‐facilitated agroinfiltration (Figure 1a). First, CRISPR‐LbCas12a was used to edit the ninth exon of *CsPDS* in Duncan plants via transient expression (Figure S1) using Xcc‐facilitated agroinfiltration (Jia and Wang, 2014b). The binary vector GFP‐p1380N‐35S‐LbCas12a‐crRNA‐cspds was constructed and agroinfiltrated into Duncan leaves (Figure 1a), which were pretreated with Xcc (Jia and Wang, 2014b). Genomic DNA that was extracted from treated Duncan leaves four days later was subjected to PCR amplification, vector ligation and colony sequencing. The sequencing results confirmed that two colonies harboured LbCas12a‐directed *CsPDS* indels among the 100 random colonies sequenced here (Figure 2). Therefore, CRISPR/LbCfp1 is functional for citrus genome editing.

### Targeted mutagenesis of EBE_PthA4_‐CsLOBP in Duncan grapefruit

**Figure 3:**
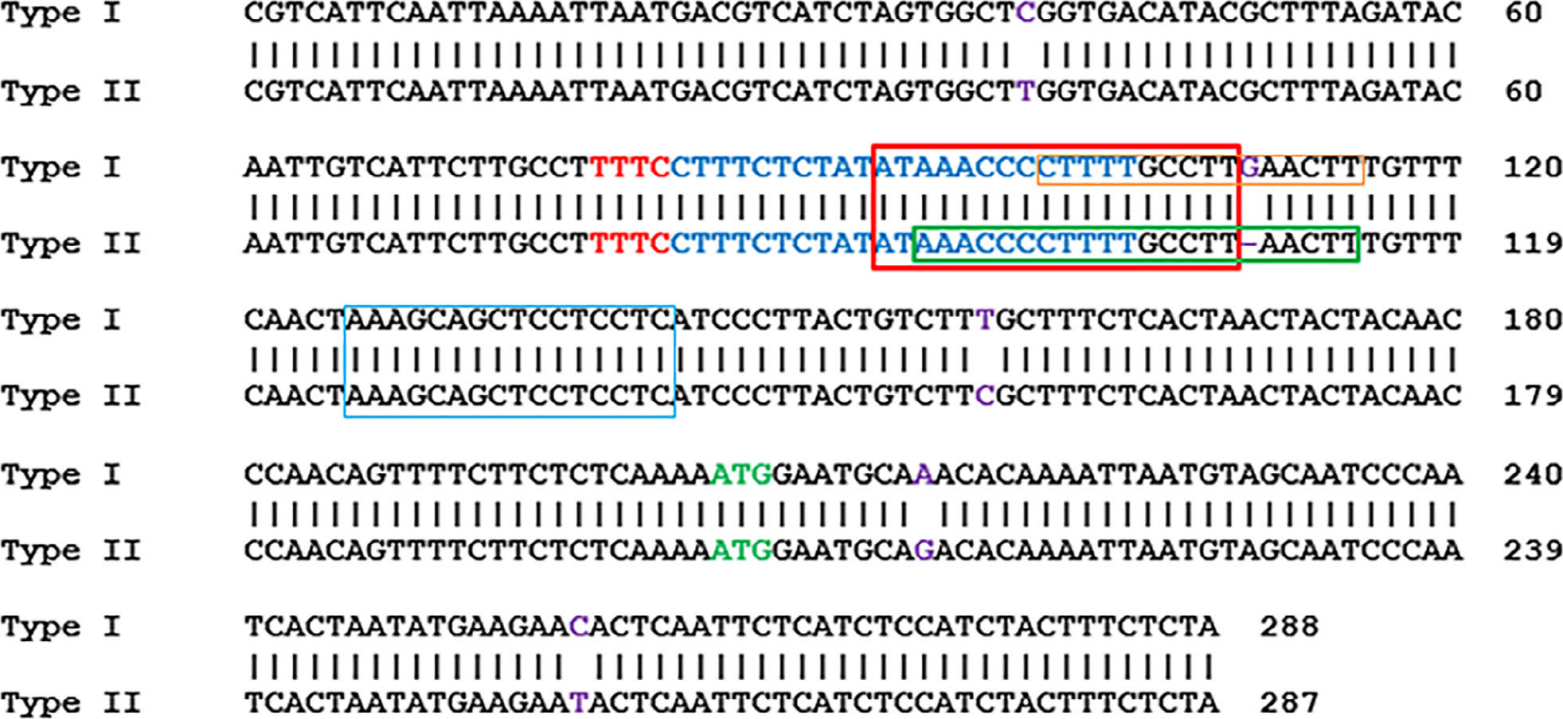
Part of the CsLOB1 and its promoter in Duncan grapefruit. A sequence alignment of two alleles of *CsLOB1*, Type I and Type II. The crRNA‐targeting site is indicated in blue. PAM is indicated in red. The translation start site is indicated in green. The difference in the two alleles is shown in purple. The EBE_P__thA4_‐CsLOBP is highlighted by a red rectangle, which is overlapped with artificial dTALE dCsLOB1.1. The dCsLOB1.2‐binding site is indicated with a blue rectangle, the dCsLOB1.3‐binding site is noted by an orange rectangle, and the artificial dTALE dCsLOB1.4 binding site is highlighted by a green rectangle.

Two binary vectors, GFP‐p1380N‐35S‐LbCas12a‐crRNA‐lobp and GFP‐p1380N‐Yao‐LbCas12a‐crRNA‐lobp, were constructed to edit EBE_PthA4_‐CsLOBP. When driven by either the CaMV35 promoter or the Yao promoter, SpCas9 was successfully used to edit the citrus genome in a previous study (Jia *et al*., 2016; Peng *et al*., 2017; Zhang *et al*., 2017). Here, we employed the CaMV 35S promoter and the Yao promoter to drive LbCas12a expression (Figure 1b and c). However, in transgenic citrus, both CaMV 35S and AtU6‐1 were successfully used to drive sgRNAs for CRISPR‐SpCas9 (Jia *et al*., 2016; Peng *et al*., 2017) and for CRISPR‐SaCas9 (Jia *et al*., 2017a). To guarantee that the crRNA could be efficiently expressed in LbCas12a‐crRNA‐lobp‐transformed citrus, both CaMV 35S and AtU6‐1 were employed to drive crRNA (Figure 1b and c). There are two types of CsLOBPs in Duncan grapefruits, Type I CsLOBP and Type II CsLOBP (Figure 3; Jia *et al*., 2016; Peng *et al*., 2017). A single crRNA was selected to target the conserved EBE_PthA4_‐CsLOBP region (Figures 1b and c, 3). By contrast, a single sgRNA could not be used to modify both types of CsLOBPs, since the sgRNA targeting region in the EBE_PthA4_‐CsLOBP contains single nucleotide polymorphisms between the two types of CsLOBPs in Duncan plants (Jia *et al*., 2016).

**Figure 4:**
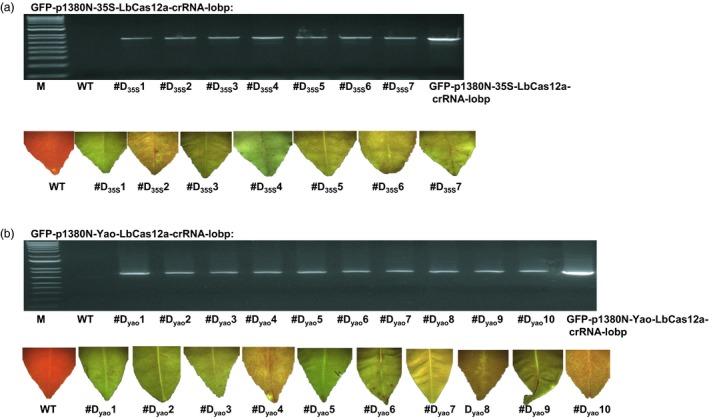
Analysis of CRISPR‐LbCas12a‐transformed Duncan grapefruit. (a) Seven GFP‐p1380N‐35S‐LbCas12a‐crRNA‐lobp‐transformed Duncan grapefruit plants (from #D_35_s1 to #D_35_s7) were evaluated by PCR analysis using the primers Npt‐Seq‐5 and 35T‐3. The plasmid GFP‐p1380N‐35S‐LbCas12a‐crRNA‐lobp was used as a positive control. The seven plants were GFP‐positive. The wild‐type grapefruit plant did not show GFP. (b) Ten GFP‐p1380N‐Yao‐LbCas12a‐crRNA‐lobp‐transformed Duncan plants (from #D_Y__ao_1 to #D_Y__ao_10) were tested by PCR analysis and GFP observation. M, 1 kb DNA ladder; WT, wild type.

The Duncan epicotyls were transformed by *Agrobacterium* cells containing the binary vector. A total of seven GFP‐p1380N‐35S‐LbCas12a‐crRNA‐lobp‐transformed Duncan plants (#D_35_s1 to #D_35_s7) were generated, and ten GFP‐p1380N‐Yao‐LbCas12a‐crRNA‐lobp transformants (#D_yao_1 to #D_yao_10) were generated. GFP fluorescence was detected in all of the transgenic plants (Figure 4a and b). Using Npt‐Seq‐5 and 35T‐3 as a pair of primers, the transgenic Duncan plants were further verified by PCR amplification (Figure 4a and b). As expected, a band measuring 750 bp was observed in transgenic plants and the positive plasmid control, whereas there was no band in the wild‐type Duncan grapefruit sample (Figure 4a and b). The results indicated that LbCas12a‐crRNA‐lobp‐transformed Duncan plants were successfully established.

### Analysis of LbCas12a‐crRNA‐lobp‐mediated indels in Duncan transformants

**Figure 5:**
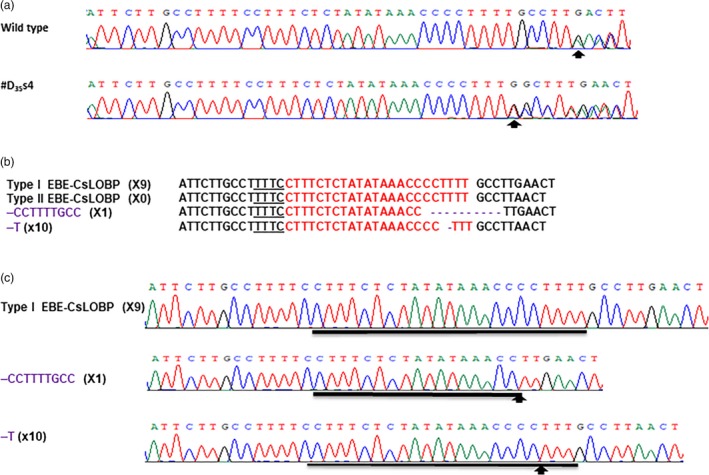
CRISPR‐LbCas12a‐mediated CsLOBP indels in transgenic Duncan #D_35_s4. (a) The chromatograms of direct PCR product sequencing. Using the primers LOBP2 and LOBP3, the CsLOBPs were amplified from wild‐type Duncan and #D_35_s4, and the CsLOB4 primer was employed for direct sequencing. The beginnings of double peaks are highlighted by arrows. (b) Targeted CsLOBP mutations directed by GFP‐p1380N‐35S‐LbCas12a‐crRNA‐lobp in transgenic Duncan #D_35_s4. The crRNA‐targeted sequence is shown in red, and the indels are highlighted in purple. (c) CRISPR‐LbCas12a‐mediated indel chromatograms in CsLOBP. Arrows are used to indicate the mutation sites.

The PCR products were sequenced directly to evaluate the LbCas12a‐crRNA‐lobp‐mediated indels in seventeen transgenic Duncan plants (Table 1). The results indicated that one transgenic Duncan line, #D_35_s4, contains changes in its chromatogram in comparison with that of the wild type (Figure 5a), whereas the other lines exhibited no changes (Table 1). It should be noted that Type I CsLOBP has one more G nucleotide next to EBE_PthA4_ than the Type II CsLOBP (Figure 3), and thus, double peaks were present from the unique guanine in wild‐type Duncan plants (Figure 5a; Jia *et al*., 2016).

**Table 1:** LbCas12a‐crRNA‐lobp‐mediated indel analysis and canker resistance of transgenic Duncan

Analysis	Lines																
	#D_35_s1	#D_35_s2	#D_35_s3	#D_35_s4	#D_35_s5	#D_35_s6	#D_35_s7	#D_yao_1	#D_yao_2	#D_yao_3	#D_yao_4	#D_yao_5	#D_yao_6	#D_yao_7	#D_yao_8	#D_yao_9	#D_yao_10
Direct sequencing of PCR products	Wild type	Wild type	Wild type	Mutant	Wild type	Wild type	Wild type	Wild type	Wild type	Wild type	Wild type	Wild type	Wild type	Wild type	Wild type	Wild type	Wild type
Sequencing of 20 random colonies	3 mutants	No mutant	No mutant	11 mutants	No mutant	No mutant	3 mutants	No mutant	No mutants	No mutant	No mutant	No mutant	No mutant	No mutant	No mutant	No mutant	No mutant
Mutation rates	15%	0	0	55%	0	0	15%	0	0	0	0	0	0	0	0	0	0
Xcc (PthA4)‐eliciting canker	Yes	Yes	Yes	Yes	Yes	Yes	Yes	Yes	Yes	Yes	Yes	Yes	Yes	Yes	Yes	Yes	Yes
dCsLOB1.4‐eliciting canker	Yes	Yes	Yes	No	Yes	Yes	Yes	Yes	Yes	Yes	Yes	Yes	Yes	Yes	Yes	Yes	Yes

Next, colony sequencing was performed to analyse CRISPR‐LbCas12a‐mediated mutations in transgenic Duncan grapefruit plants. Among the 20 colonies sequenced for each transgenic line, no mutations were observed in all the Yao‐LbCas12a‐transformed Duncan plants and four 35S‐LbCas12a‐transformed lines (#D_35_s2, #D_35_s3, #D_35_s5 and D_35_s6; Table 1), whereas #D_35_s1, #D_35_s4 and #D_35_s7 contained indels (Figure 5b and c, Figure S2). The mutation rates of #D_35_s1, #D_35_s4 and #D_35_s7 were 15%, 55% and 15%, respectively (Table 1). The Type II EBE‐CsLOBPs were 100% mutated according to Figure 5. All the mutation genotypes were deletions (Figure 5b and c, Figure S2). Specifically, the deletion of one thymine took place only in Type II EBE‐CsLOBP among the sequenced colonies, whereas a longer deletion occurred only on Type I EBE‐CsLOBP (Figure 5b and c, Figure S2). Most importantly, as expected, both Type I EBE‐CsLOBP and Type II EBE‐CsLOBP were readily modified by the single crRNA‐targeting sequence (Figure 5b and c, Figure S2).

### #D_35_s4 transgenic plant alleviating XccΔpthA4:dCsLOB1.4 infection

Seventeen transgenic Duncan plants were treated with Xcc at a concentration of 5 × 10^8^ CFU/mL. Canker symptoms were observed in all transgenic lines, similar to the wild‐type control plants, at 5 days post‐inoculation (DPI; Table 1). The results are consistent with those of a previous study, in which canker could readily develop on Cas9/sgRNA:CsLOBP1‐transformed Duncan plants harbouring one intact CsLOBP allele (Jia *et al*., 2016).

**Figure 6:**
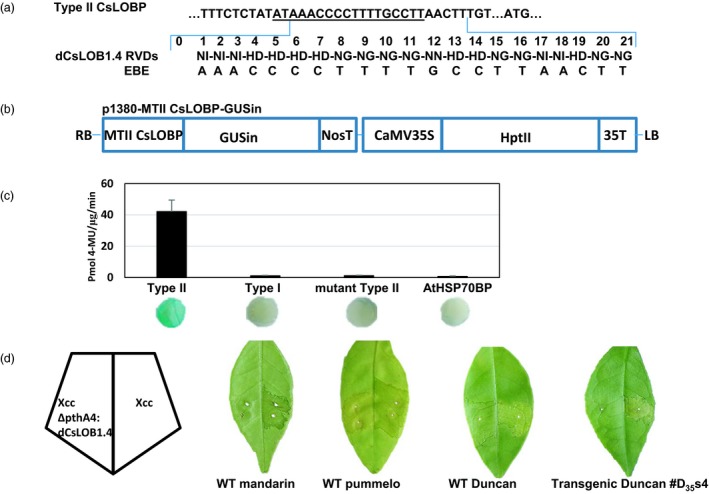
Transgenic Duncan #D_35_s4 resistant against Xcc306ΔpthA4:dCsLOB1.4. (a) Artificial dTALE dCsLOB1.4 was developed to activate Type II CsLOBP specifically. RVDs of the artificial dTALE dCsLOB1.4 bind to AAACCCCTTTTGCCTTAACTT, 2 bp downstream of EBE_pthA4_‐TII CsLOBP, which is underlined. (b) A schematic diagram of p1380‐MTII CsLOBP‐GUSin. MTII CsLOBP, mutant Type II CsLOBP; GUSin, the intron‐containing β‐glucuronidase; and HptII, the coding sequence of hygromycin phosphotransferase II. (c) Via Xcc306ΔpthA4:dCsLOB1.4‐facilitated agroinfiltration, a quantitative GUS assay and GUS histochemical staining were used to study the effects of Xcc‐derived dCsLOB1.4 on CsLOBPs. Notably, only under the control of Type II CsLOBP could GUS expression be activated. The experiments were repeated twice. (d) Five days post‐Xcc inoculation, citrus canker symptoms were observed on mandarin (containing Type I CsLOBP), pummelo (containing Type II CsLOBP), Duncan grapefruit (containing Type I CsLOBP and Type II CsLOBP) and transgenic Duncan #D_35_s4 (containing Type I CsLOBP and mutant Type II CsLOBP) grapefruit, since the PthA4 derived from Xcc could activate Type I CsLOBP and Type II CsLOBP. Five days after Xcc306ΔpthA4:dCsLOB1.4 treatment, citrus canker symptoms were not observed on mandarin and transgenic Duncan #D_35_s4, since dCsLOB1.4 could not activate Type I CsLOBP and mutant Type II CsLOBP.

In our previous study, dCsLOB1.1 and dCsLOB1.2 were developed to activate two types of CsLOBPs (Hu *et al*., 2014). The dCsLOB1.1 binding site is 5′TAAAGCAGCTCCTCCTC3′, and the dCsLOB1.2 recognition sequence is 5′TATAAACCCCTTTTGCCTT3′ (Figure 3). Later, dCsLOB1.3 was built to recognize the Type I EBE‐CsLOBP allele only, the binding sequence of which is 5′CCTTTTGCCTTGAACTTT3′ (Figure 3; Jia *et al*., 2016). Two Cas9/sgRNA:CsLOBP1‐transformed lines with the highest mutation rate for the Type I EBE‐CsLOBP allele could resist XccΔpthA4: dCsLOB1.3 (Jia *et al*., 2016). Here, we constructed a novel dTALE, dCsLOB1.4 (Figure 6a), the repeat variable di‐residues (RVDs) of which specifically bind to the 21‐nucleotide sequence 5′TAAACCCCTTTTGCCTTAACTT3′ in the Type II CsLOBP (Figures 3, 6a), whereas one extra ‘G’ nucleotide is present in the Type I CsLOBP and one ‘T’ nucleotide is absent from the mutated Type II CsLOBP compared to the wild‐type II CsLOBP.

The designed TALE dCsLOB1.4 was developed here to specifically activate Type II EBE‐CsLOBP (Figure 6a), but not the Type I CsLOBP and mutant Type II CsLOBP. To confirm dCsLOB1.4‐specific recognition, the binary vectors p1380‐AtHSP70BP‐GUSin, p1380‐TI CsLOBP‐GUSin, p1380‐TII CsLOBP‐GUSin and p1380‐MTII CsLOBP‐GUSin (Figure 6b) were used to perform an XccΔpthA4:dCsLOB1.4‐facilitated agroinfiltration. It should be noted that p1380‐AtHSP70BP‐GUSin was used as a negative control (Jia and Wang, 2014b). As expected, only Type II CsLOBP‐driven GUS expression could be specifically activated (Figure 6c), whereas neither MTII CsLOBP‐GUSin nor TI CsLOBP‐GUSin was activated (Figure 6c). The results indicated that dCsLOB1.4 specifically recognizes the Type II CsLOBP. The mandarin has two Type I EBE‐CsLOBP alleles, and the pummelo contains two Type II EBE‐CsLOBP alleles (Wu *et al*., 2014). In the presence of XccΔpthA4:dCsLOB1.4, canker symptoms develop on pummelo but not on mandarin (Figure 6d). The results further confirmed that, as expected, dCsLOB1.4 specifically activates Type II EBE‐CsLOBP, resulting in canker on pummelo. After XccΔpthA4:dCsLOB1.4 infection, #D_35_s4 showed alleviated XccΔpthA4:dCsLOB1.4 infection owing to its 100% mutation on Type II EBE‐CsLOBP (Figures 5b, c and 6d, Table 1), whereas canker symptoms were observed in other transgenic Duncan lines (Table 1).

### Potential off‐targets of LbCas12a‐crRNA‐lobp

Based on the sweet orange genome, the potential off‐targets generated by LbCas12a‐crRNA‐lobp were analysed (mismatch number = 2, RNA bulge size = 1) using a web tool (http://www.rgenome.net/cas-offinder/; Bae *et al*., 2014). All the potential off‐targets using 1 or 2 mismatches are located at the LbCas12a‐crRNA‐lobp‐targeting site (Figure S3), indicating that there might be no potential off‐targets of LbCas12a‐crRNA‐lobp. However, we could not totally rule out the possibility that there could be mutations in potential off‐targets with three or more mismatches.

## Discussion

For the first time, we demonstrated that a citrus genome could be modified specifically using either the transient expression of LbCas12a via Xcc‐facilitated agroinfiltration or the constitutive expression of LbCas12a in transgenic Duncan plants.

All of the mutations generated by CRISPR‐LbCas12a in citrus were deletions. In the LbCas12a‐mediated mutations of *CsPDS* and Type I CsLOBP, the indels were relatively long deletions, which are consistent with those in other plants (Begemann *et al*., 2017; Endo *et al*., 2016; Ferenczi *et al*., 2017; Hu *et al*., 2017; Kim *et al*., 2017; Tang *et al*., 2017; Wang *et al*., 2017a,b,c; Xu *et al*., 2016; Yin *et al*., 2017). The longer deletions could be attributable to 5′ overhangs resulting from the stagger cutting of Cas12a at sites distal to the PAM (Tang *et al*., 2017; Zetsche *et al*., 2015). Interestingly, all of the Type II CsLOBP mutations generated by LbCas12a were 1 bp deletions among the colonies that were sequenced (Figure 5b and c, Figure S2A), and they are similar to the short indels (1–2 bp) induced by SpCas9 in citrus (Jia *et al*., 2016, 2017b; Peng *et al*., 2017; Zhang *et al*., 2017).

The mutation frequencies of #D_35_s1, #D_35_s4 and #D_35_s7 were 15%, 55% and 15%, respectively. The average mutant rate in #D_35_s1, #D_35_s4 and #D_35_s7 was 28.3%, which is similar to that of FnCas12a‐transformed tobacco (28.2%), but lower than that of FnCas12a‐transformed rice (47.2%; Endo *et al*., 2016). The authors suggested that the different processes, which were employed to develop transgenic tobacco and rice, might be the cause for the different mutation frequencies (Endo *et al*., 2016). Additionally, a low mutation efficacy of 2% was observed for citrus when LbCas12a transient expression was used. The mutation frequencies induced by the transient expression of AsCas12a ranged from 0.6% to 10%, whereas the mutation frequencies mediated by LbCas12a ranged from 15% to 25% in rice (Tang *et al*., 2017). A nearly 100% biallelic mutation efficiency was observed for LbCas12a‐mediated genome editing in rice, whereas the biallelic mutation efficiency from LbCas12a was only 5% in citrus. The lower mutation efficacy from LbCas12a in citrus might result from the different crRNA designs that were used (Tang *et al*., 2017). This finding signifies the need to improve the efficacy of LbCas12a‐mediated genome editing.

Unexpectedly, no indels were detected in the Yao‐LbCas12a‐transformed Duncan plants. The Yao promoter was used to drive SpCas9 expression for high‐efficiency citrus genome editing in a previous report (Zhang *et al*., 2017). There are some differences between the two studies. In the previous study, Carrizo was transformed by Yao‐SpCas9, and sgRNAs were driven by AtU6‐26 (Zhang *et al*., 2017), whereas Duncan grapefruit was transformed by Yao‐LbCas12a, and AtU6‐1 and CaMV 35S were employed to drive crRNA (Figure 1c). It remains unclear whether these differences affect the mutation efficiency induced by SpCas9 and LbCas12a. More work is needed to clarify whether the Yao promoter is suitable for driving the Cas12a. Interestingly, heat treatment has been used to enhance genome editing in Cas9‐transformed citrus (LeBlanc *et al*., 2018). It is worth testing whether heat shocks can enhance the mutations in LbCas12a‐transformed citrus. In addition, several novel strategies, including the ribozyme processing strategy (Tang *et al*., 2017), the crRNA processing strategy (Wang *et al*., 2018) and the single transcript unit strategy (Tang *et al*., 2018; Xu *et al*., 2018), have been developed to enhance plant genome editing. Undoubtedly, these strategies might help to optimize the efficacy of citrus genome editing in future.

Intriguingly, CRISPR‐LbCas12a ribonucleoproteins (RNPs) have already been used to edit the genomes to generate transgene‐free mutations in soybeans and tobacco (Kim *et al*., 2017). The delivery of CRISPR‐LbCas12a RNPs bypasses the need to develop a system for removing foreign DNAs from genetically modified plants. It would be worth testing whether Cas12a RNPs could be harnessed to generate foreign DNA‐free genome‐modified citrus.

In summary, we presented our recent progress in using CRISPR‐LbCas12a to edit a citrus genome via Xcc‐facilitated agroinfiltration and stable transformation. Because of its unique targeted mutagenesis features, CRISPR‐LbCas12a can undoubtedly enhance the scope and specificity of citrus genome editing, which was supported by this study. To enhance the scope of citrus genome editing, we successfully used single crRNA targeting to modify two alleles of EBE_PthA4_‐CsLOBPs. Therefore, CRISPR‐LbCas12a should be regarded as a powerful complementary tool for citrus genome engineering, in addition to CRISPR‐SpCas9 and CRISPR‐SaCas9 (Jia and Wang, 2014a; Jia *et al*., 2016, 2017a,b; Peng *et al*., 2017; Zhang *et al*., 2017).

## Materials and methods

### Plasmid construction

The CaMV 35S promoter was amplified using the primers CaMV35‐5‐*Sbf*I (5′‐AGGTCCTGCAGGTCCCCAGATTAGCCTTTTCAATTT‐3′) and CaMV35‐3‐*Kpn*I‐*Bam*HI (5′‐AGGTGGATCCGGTACCTATCGTTCGTAAATGGTGAAAATT‐3′) and then cloned into *Sbf*I‐*Bam*HI‐digested GFP‐p1380N‐Cas9 to produce GFP‐p1380N‐*Kpn*I‐Cas9. GFP‐p1380N‐Cas9 was constructed in a previous study (Jia *et al*., 2017a). LbCas12a harbouring a nuclear localization signal (NLS) and an HA tag at its C‐terminus was obtained from Addgene plasmid pY016 after a *Kpn*I and *Eco*RI cut (Zetsche *et al*., 2015). The *Kpn*I‐LbCas12a‐*Eco*RI fragment was inserted into *Kpn*I‐*Eco*RI‐cut GFP‐p1380N‐KpnI‐Cas9 to generate GFP‐p1380N‐35S‐LbCas12a. By using *Arabidopsis* genomic DNA as a template, the Yao promoter was amplified with a pair of primers, Yao‐5‐*Sbf*I (5′‐AGGTCCTGCAGGATGGGAAATTCATTGAAAACCCT‐3′) and Yao‐3‐*Kpn*I (5′‐AGGTGGTACCGGATCCTTTCTTCTTCTCGTTGTTGTACTTCAT‐3′). The *Sbf*I‐*Kpn*I‐digested Yao promoter was cloned into *Sbf*I‐*Kpn*I‐cut GFP‐p1380N‐35S‐LbCas12a to obtain GFP‐p1380N‐Yao‐LbCas12a. The Nos terminator (NosT) was amplified using NosT‐5‐*Eco*RI (5′‐AGGATCCACCGGTGCACGAATTCCGAATTTCCCCGATCGTTCAA‐ 3′) and NosT‐3‐*Xho*I‐*Asc*I‐*Xba*I‐*Pme*I (5′‐AGTTTAAACTCTAGACAAGGCGCGCCATTTAAATCTCGAG CCGATCTAGTAACATAGATGACAC‐3′). After *Eco*RI digestion, NosT was inserted into *Eco*RI‐*Sfo*I‐digested pUC18 to generate pUC‐NosT‐MCS.

From p1380N‐sgRNA (Jia *et al*., 2017a), the CaMV 35S promoter was amplified using the primers CaMV35‐5‐*Xho*I (5′‐ACTCGAGACTAGTACCATGGTGGACTCCTCTTAA‐3′) and CaMV35‐crRNA‐3 (5′‐phosphorylated CTACACTTAGTAGAAATTCCTCTCCAAATGAAA TGAACTTCCT‐ 3′), and the crRNA‐cspds‐NosT fragment was amplified using the primers crRNA‐cspds‐P (5′‐phosphorylated‐ATAGGTAACTGAAGCTTGAGGATATGAATTTCCCCGA TCGTTCAAACATTTG‐3′) and NosT‐3‐*Asc*I (5′‐ACCTGGGCCCGGCGCGCCGATCTAGT AACATAGATGA‐3′). Through three‐way ligation, *Xho*I‐cut CaMV35S and *Asc*I‐digested crRNA‐cspds‐NosT were inserted into *Xho*I‐*Asc*I‐treated pUC‐NosT‐MCS to build pUC‐NosT‐crRNA‐cspds. Subsequently, the *Eco*RI‐NosT‐crRNA‐cspds‐NosT‐*Pme*I fragment was cloned into *Eco*RI‐*Pme*I‐cut GFP‐p1380N‐35S‐LbCas12a to construct GFP‐p1380N‐35S‐LbCas12a‐crRNA‐cspds (Figure 1a), which was designed to edit the sequence located 15 641 bp downstream of the ATG in *CsPDS* (Figure S1).

Similarly, the CaMV 35S promoter was PCR‐amplified using the primers CaMV35‐5‐*Xho*I and CaMV35‐crRNA‐3, and the crRNA1‐lobp‐NosT was PCR‐amplified using the primers crRNA‐lobp‐P (5′‐phosphorylated‐ ATCTTTCTCTATATAAACCCCTTTTGAATTTCCCCG ATCGTTCAAACATTTG‐3′) and NosT‐3‐*Asc*I. *Xho*I‐cut CaMV35S and *Asc*I‐digested crRNA‐lobp‐NosT were inserted into *Xho*I‐*Asc*I‐cut pUC‐NosT‐MCS to build pUC‐NosT‐35S‐crRNA‐lobp through three‐way ligation. With GFP‐p1380N‐SaCas9/35S‐sgRNA1:AtU6‐sgRNA2 as a template (Jia *et al*., 2017a), the AtU6‐1 was amplified using AtU6‐1‐5‐*Asc*I (5′‐AGGT GGCGCGCCTCTTACAGCTTAGAAATCTCAAA‐3′) and AtU6‐1‐crRNA‐3 (5′‐phosphorylated‐CTACACTTAGTAGAAATTCAATCACTACTTCGTCTCTAACCATATA‐3′). Using crRNA‐lobp‐P and NosT‐3‐*Spe*I (5′‐AGGTACTAGTCCGATCTAGTAACATAGA TGACA‐3′), the crRNA2‐lobp‐NosT fragment was amplified. Through three‐way ligation, *Asc*I‐cut AtU6‐1 and *Spe*I‐digested crRNA2‐lobp‐NosT were inserted into *Asc*I‐*Xba*I‐treated pUC‐NosT‐35S‐crRNA‐lobp to form pUC‐NosT‐crRNA‐lobp. Finally, the *EcoR*I‐NosT‐35S‐crRNA‐lobp‐NosT‐AtU6‐1‐crRNA‐lobp‐NosT‐*Pme*I fragment was cloned into *Eco*RI‐*Pme*I‐cut GFP‐p1380N‐35S‐LbCas12a to construct GFP‐p1380N‐35S‐LbCas12a‐crRNA‐lobp (Figure 1b) or into *Eco*RI‐*Pme*I‐cut GFP‐p1380N‐Yao‐LbCas12a to form GFP‐p1380N‐Yao‐LbCas12a‐crRNA‐lobp (Figure 1c).

Using forward primer LOBP1 and reverse primer LOBP2 (Jia *et al*., 2016), the mutant Type II CsLOBP, which contains a thymine deletion, was amplified from transgenic line #D_35_s4. After sequencing, the *Hin*dIII‐*Bam*HI‐digested PCR fragment was inserted into *Hin*dIII‐*Bam*HI‐treated p1380‐35S‐GUSin to form binary vectors p1380‐MTII CsLOBP‐GUSin (Figure 6b). Binary vectors p1380‐AtHSP70BP‐GUSin, p1380‐TI CsLOBP‐GUSin and p1380‐TII CsLOBP‐GUSin were developed previously (Jia *et al*., 2016).

Through the electroporation method, the binary vectors were introduced into *Agrobacterium tumefaciens* strain EHA105. Recombinant *Agrobacterium* cells were cultivated for Xcc‐facilitated agroinfiltration or epicotyl citrus transformation.

### Xcc‐facilitated agroinfiltration in Duncan grapefruit

The Duncan grapefruit (*Citrus paradisi*) was grown in a greenhouse at approximately 27 °C and then pruned for uniform shooting before Xcc‐facilitated agroinfiltration.

As described before (Jia and Wang, 2014b), the Duncan leaves were first inoculated with a culture of actively growing Xcc, which was resuspended in sterile tap water at a concentration of 5 × 10^8^ CFU/mL. Twenty‐four hours later, the leaf areas, which were pretreated with XccΔgumC, were subjected to agroinfiltration with recombinant *Agrobacterium* cells harbouring GFP‐p1380N‐35S‐LbCas12a‐crRNA‐cspds. Four days after the agroinfiltration, the genomic DNA was extracted from the treated leaves. Similarly, the XccΔpthA4:dCsLOB1.4‐treated leaf areas were agroinfiltrated with recombinant *Agrobacterium* containing p1380‐TI CsLOBP‐GUSin, p1380‐TII CsLOBP‐GUSin, p1380‐MTII CsLOBP‐GUSin or p1380‐AtHSP70BP‐GUSin. Four days later, the leaves were collected for a GUS assay.

### *Agrobacterium*‐mediated Duncan grapefruit transformation

A citrus transformation was performed as reported before (Jia *et al*., 2017b). In summary, Duncan epicotyl explants were coincubated with recombinant *Agrobacterium* cells harbouring a binary vector, with either GFP‐p1380N‐35S‐LbCas12a‐crRNA‐lobp or GFP‐p1380N‐Yao‐LbCas12a‐crRNA‐lobp. Five weeks later, all the explants were inspected for GFP fluorescence. Later, GFP‐positive sprouted shoots were micrografted onto ‘Carrizo’ citrange rootstock plants [*C. sinensis* (L.) Osbeck × *Poncirus trifoliata* (L.) Raf.] for continuous cultivation and further analysis.

The transgenic plants were subjected to PCR analysis with a pair of primers, Npt‐Seq‐5 (5′‐TGTGCTCGACGTTGTCACTGAAGC‐3′) and 35T‐3 (5′‐TTCGGGGGATCTGGATTT TAGTAC‐3′).

### PCR amplification of mutagenized *CsPDS* and CsLOBP

Genomic DNA was extracted from the Duncan leaves that were treated with Xcc‐facilitated agroinfiltration or each transgenic Duncan line.

To test the GFP‐p1380N‐35S‐LbCas12a‐crRNA‐cspds‐mediated indels in the *CsPDS* gene, PCR was performed using the primers CsPDS‐5‐P7 (5′‐TGGCAATGTGATTGACGGAGATGC‐3′) and CsPDS‐3‐P8 (5′‐ATGAGTCCTCCTTGTTACTTCAGT‐3′), which flanked the targeted site of *CsPDS*. The template was genomic DNA, which was extracted from Duncan leaves treated with GFP‐p1380N‐35S‐LbCas12a‐crRNA‐cspds. Using blunt‐end cloning, the *CsPDS* PCR products were ligated into a PCR Blunt II‐TOPO vector (Life Technologies, Carlsbad, CA). A total of one hundred random colonies were chosen for DNA sequencing. A Chromas Lite program was employed to analyse the sequencing results.

To analyse the LbCas12a‐crRNA‐lobp‐mediated CsLOBP mutations, PCR was performed using a pair of primers, LOBP3 (5′‐AGGTAAGCTTATTCATATTAACGTTATCAATGATT‐3′) and LOBP2 (5′‐ACCTGGATCCTTTTGAGAGAAGAAAACTGTTGGGT‐3′; Jia *et al*., 2016). Following purification, the PCR products were subjected to either ligation or direct PCR product sequencing using the primer CsLOB4 (5′‐CGTCATTCAATTAAAATTAATGAC‐3′). After transformation, 20 random colonies for each transgenic Duncan line were chosen for detailed sequencing. The sequencing results were further analysed using the Chromas Lite program.

### GFP detection

A Zeiss Stemi SV11 dissecting microscope (Thornwood, NY) equipped with an Omax camera was used to study the GFP‐p1380N‐35S‐LbCas12a‐crRNA‐lobp‐transformed and GFP‐p1380N‐Yao‐LbCas12a‐crRNA‐lobp‐transformed Duncan plants under illumination by a Stereo Microscope Fluorescence Adapter (NIGHTSEA). Subsequently, the transgenic plant leaves were photographed with Omax ToupView software.

### GUS assay

Four days after the Xcc‐facilitated agroinfiltration, the histochemical staining of GUS and a quantitative GUS assay were performed on the treated citrus leaves as described previously (Jia and Wang, 2014b).

### Canker symptom assay in citrus

All the citrus plants were grown in a greenhouse. Prior to the canker pathogen inoculation, the Duncan grapefruit (*Citrus paradisi*), pummelo (*C. maxima*), Willowleaf mandarin (*Citrus reticulata*) and transgenic Duncan grapefruit plants were pruned to promote shooting. With a needleless syringe, the same aged leaves were inoculated with Xcc or XccΔpthA4:dCsLOB1.4, which were resuspended in sterile tap water (5 × 10^8^ CFU/mL). The ensuing canker development was observed and photographed at different time points.

## Conflict of interest

The authors declare no conflict of interest.

## Supporting information


**Figure S1** Schematic map of *CsPDS*.
**Figure S2** CRISPR‐LbCas12a‐mediated CsLOBP indels in transgenic Duncan #D_35_s1 and #D_35_s7.
**Figure S3** Potential off‐targets of LbCas12a‐crRNA‐lobp in transgenic Duncan grapefruit.
