# Supplementary material for: CRISPR‐LbCas12a‐mediated modification of citrus
Source: Plant Biotechnol J. 2019 Apr 10;17(10):1928–37. doi: 10.1111/pbi.13109 (PMC6737016; doi:10.1111/pbi.13109)
Supplement: Supplementary file 1 — Figure S1 Schematic map of CsPDS. Figure S2 CRISPR‐LbCas12a‐mediated CsLOBP indels in transgenic Duncan #D35s1 and #D35s7. Figure S3 Potential off‐targets of LbCas12a‐crRNA‐lobp in transgenic Duncan grapefruit. [file PBI-17-1928-s001.pdf]

Supplementary Figure 1

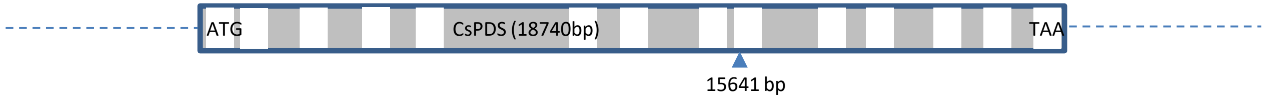

Supplementary Figure 1. Schematic map of *CsPDS*. The *CsPDS*-targeting crRNA is located in the ninth exon of *CsPDS*, 15641 bp downstream of *CsPDS* ATG. The intron parts were indicated by gray.

Supplementary Figure 2

(A) LbCas12a-crRNA-lobp-mediated indels and their chromatograms in #$D_{35s}1$

Type I EBE-CsLOBP (X10)	ATTCTTGCCTTTTCCTTTCTCTATATAAACCCCTTTT GCCTTGAACT
Type II EBE-CsLOBP (X7)	ATTCTTGCCTTTTCCTTTCTCTATATAAACCCCTTTT GCCTTAACT
–CC (X1)	ATTCTTGCCTTTTCCTTTCTCTATATAAA - - CCTTTT GCCTTGAACT
–T (x2)	ATTCTTGCCTTTTCCTTTCTCTATATAAACCCC -TTT GCCTTAACT

Type I EBE-CsLOBP (X10)

Type II EBE-CsLOBP (X7)

–CC (X1)

–T (x2)

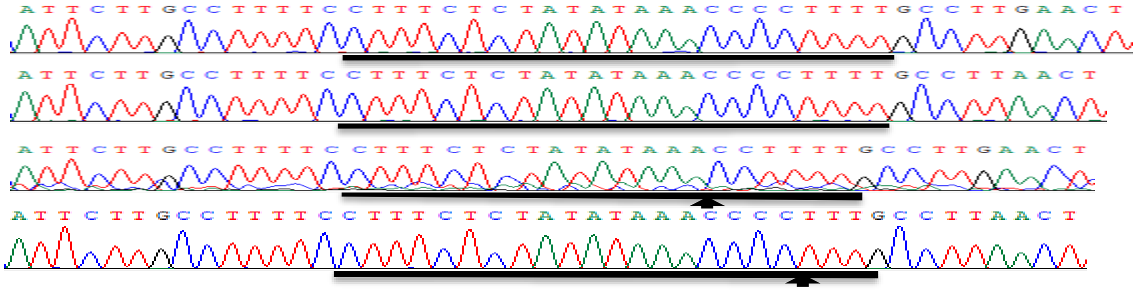

(B) LbCas12a-crRNA-lobp-mediated indels and their chromatograms in #$D_{35s}7$

Type I EBE-CsLOBP (X9)	ATTCTTGCCTTTTCCTTTCTCTATATAAACCCCTTTT GCCTTGAACT
Type II EBE-CsLOBP (X8)	ATTCTTGCCTTTTCCTTTCTCTATATAAACCCCTTTT GCCTTAACT
–AACCCCTTTTGCC (X1)	ATTCTTGCCTTTTCCTTTCTCTATATA - - - - - - - - - - - - - TTGAACT
–CCCCTTTT, +1G (x1)	ATTCTTGCCTTTTCCTTTCTCTATATAAA - - - - - - - -GGCCTTGAACT
–CTATATAAACCCCTTTTG, +GTA (X1)	ATTCTTGCCTTTTCCTTTCT- - - - - - - - - - - - - - - - - -GTA CCTTGAACT

Type I EBE-CsLOBP (X9)

Type II EBE-CsLOBP (X8)

–AACCCCTTTTGCC (X1)

–CCCCTTTT, +1G (x1)

–CTATATAAACCCCTTTTG, +GTA (X1)

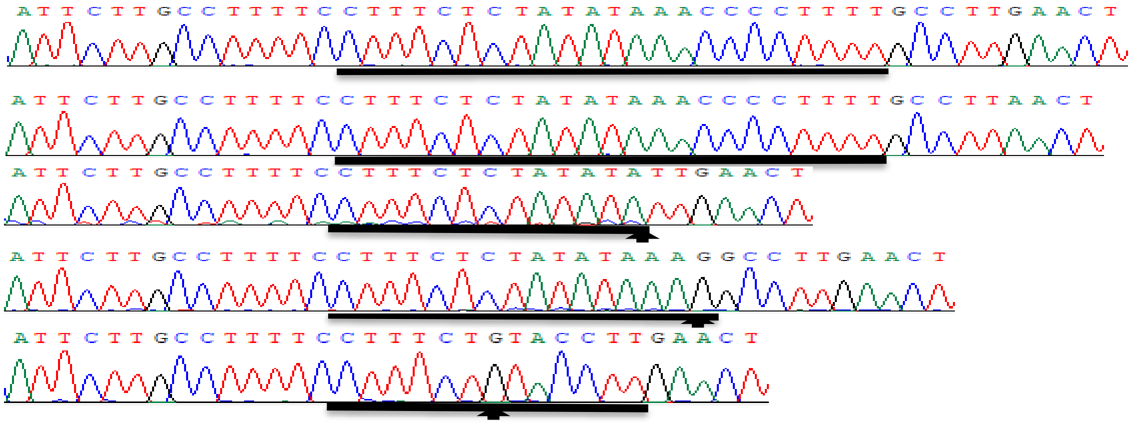

Supplementary Figure 2. CRISPR-LbCas12a-mediated CsLOBP indels in transgenic Duncan #D_{35}s1 and #D_{35}s7. Targeted CsLOBP mutations in GFP-p1380N-35S-LbCas12a-crRNA-lobp-transformed Duncan lines #D_{35}s1 (A) and #D_{35}s1 (B).

Bulge Type	Target	Chromosome	Position	Direction	Mismatches	Bulge Size
RNA	crRNA: TTTVCTTTCTCTATATAAACCCCTTTT<br>DNA: TTTCCTTTCTCTATATAAACCCC-TTT	NC_023052.1	28359398	-	0	1
RNA	crRNA: TTTVCTTTCTCTATATAAACCCCTTTT<br>DNA: TTTCCTTTCTCTATATAAACCCCT-TT	NC_023052.1	28359398	-	0	1
RNA	crRNA: TTTVCTTTCTCTATATAAACCCCTTTT<br>DNA: TTTCCTTTCTCTATATAAACCCCTT-T	NC_023052.1	28359398	-	0	1
X	crRNA: TTTVCTTTCTCTATATAAACCCCTTTT<br>DNA: TTTCCTTTCTCTATATAAACCCCTTTT	NC_023052.1	28359397	-	0	0
RNA	crRNA: TTTVCTTTCTCTATATAAACCCCTTTT<br>DNA: TTTCCTTTCTCTATAT-AAaCCCcTTT	NC_023052.1	28359398	-	2	1
RNA	crRNA: TTTVCTTTCTCTATATAAACCCCTTTT<br>DNA: TTTCCTTTCTCTATATA-AaCCCcTTT	NC_023052.1	28359398	-	2	1
RNA	crRNA: TTTVCTTTCTCTATATAAACCCCTTTT<br>DNA: TTTCCTTTCTCTATATAA-aCCCcTTT	NC_023052.1	28359398	-	2	1
RNA	crRNA: TTTVCTTTCTCTATATAAACCCCTTTT<br>DNA: TTTCCTTTCTCTATATAAA-CCCcTTT	NC_023052.1	28359398	-	1	1
RNA	crRNA: TTTVCTTTCTCTATATAAACCCCTTTT<br>DNA: TTTCCTTTCTCTATATAAAC-CCcTTT	NC_023052.1	28359398	-	1	1
RNA	crRNA: TTTVCTTTCTCTATATAAACCCCTTTT<br>DNA: TTTCCTTTCTCTATATAAACC-CcTTT	NC_023052.1	28359398	-	1	1
RNA	crRNA: TTTVCTTTCTCTATATAAACCCCTTTT<br>DNA: TTTCCTTTCTCTATATAAACCC-cTTT	NC_023052.1	28359398	-	1	1

Supplementary Figure 3. Potential off-targets of LbCas12a-crRNA-lobp in transgenic Duncan grapefruit. A web software, whose link is http://www.rgenome.net/cas-offinder/, was employed to analyze off-targets induced by LbCas12a-crRNA-lobp in transgenic Duncan. The potential off-targets were actually located to the same sequence as the LbCas12a-crRNA-lobp-targeting site, marked by blue arrows. Therefore, there was no potential off-targets.
